# Allergic reactions to emerging food allergens in Canadian children

**DOI:** 10.1186/s13223-021-00573-y

**Published:** 2021-07-13

**Authors:** Lianne Soller, Sebastien La Vieille, Scott B. Cameron, Raymond Mak, Victoria E. Cook, Jennifer Gerdts, Edmond S. Chan

**Affiliations:** 1 Division of Allergy and Immunology, Department of Pediatrics, Faculty of Medicine, University of British Columbia, 4480 Oak St, Rm 1C31B, Vancouver, BC V6H 3V4 Canada; 2 Food Directorate, Health Canada, Ottawa, ON Canada; 3 Victoria Allergy, Victoria, BC Canada; 4 Food Allergy Canada, Toronto, ON Canada

**Keywords:** Emerging allergens, Legume allergy, Seed allergy, Fruit and vegetable allergy

## Abstract

Most Canadian food allergy data has focused on Health Canada’s priority food allergens. This study describes which non-priority (emerging) food allergens were most commonly reported by Canadian parents and categorized/confirmed by allergists. A secondary aim was to describe severity of allergic reactions to emerging allergens. Parents reported allergic reactions to emerging food allergens experienced by their child (< 18 years) which occurred in the past 12 months, and allergists categorized/confirmed them according to likelihood of IgE-mediated food allergy. Of 68 eligible patients completing the survey, the most commonly reported emerging allergens were fruits/vegetables (58.8%), seeds (22.1%), legumes (19.1%) and other (11.8%). Median allergist ranking for legumes was ‘probable’ IgE-mediated food allergy, ‘possible’ for seeds and fruits/vegetables, and ‘unlikely’ for other. Median reaction severity was mild for legumes, and moderate for seeds, fruits/vegetables, and other. Our study highlights that non-priority food allergens, namely legumes and seeds, can lead to probable/likely allergic reactions in Canadian children. These food allergens are increasing in popularity in the Canadian diet, which could lead to increasing reports of allergic reactions. More research is needed to confirm reports of reactions to emerging allergens, and to document their inclusion as ingredients in packaged foods.

## Main text

In 2018, our group published the first case report of lupin allergy in Canada [1]. This case prompted us to begin discussions with Health Canada on how to collect data on allergic reactions to foods beyond their 11 priority food allergens (i.e., those known to be most frequently associated with food allergies and allergic reactions). Our primary aim was to determine which other foods not on this list, hereafter referred to as “emerging food allergens,” were most commonly reported by Canadian parents and categorized/confirmed by allergists. A secondary aim was to describe severity of allergic reactions to emerging allergens.

An online survey was distributed through Food Allergy Canada, a national parent advocacy group, which asked about allergic symptoms, timing of symptoms after exposure, and duration of symptoms to determine whether the reaction was IgE-mediated.

Eligibility for the survey included:

1. Allergic reaction to food OUTSIDE of 11 priority allergens,
2. Allergic reaction ≤ 12 months ago,
3. Child ≤ 18 years, and,
4. Food causing reaction made/packaged and purchased in Canada.

Two board-certified allergists independently reviewed parent surveys and categorized likelihood of IgE-mediated food allergy as “Not/Not enough information”, “Unlikely”, “Possible”, or “Probable/Likely”, based on parent responses. Each patient was then assigned one likelihood estimate calculated as the least certain (lowest likelihood) of the two allergist estimates. Severity of allergic reaction was graded 1–4 based on modified WAO criteria [2].

Descriptive statistics were calculated for all variables. Two-sample t-tests were used to determine whether there was a statistical association (p < 0.05) between: (1) Age (< 6 vs. ≥ 6 years) and food allergen type (legumes (not including peanut), seeds (not including sesame), fruits/vegetables, other), (2) epinephrine use and food allergen type, and (3) epinephrine use and grade of allergic reaction. Data were analyzed using Stata SE 15. Ethics approval was waived as this was a quality improvement study.

Between Jun/2019 and Sept/2020, 471 parents clicked on the survey, of which 145 answered the eligibility questions (Response rate: 30.8%). Of these 145, 77 did not meet eligibility criteria. Of 68 remaining eligible children, about half (48.5%) were female, with a relatively equal age distribution. (Table 1) Many children were diagnosed with other food allergies (82.4%).

**Table 1:** Demographics of eligible children

	N (%)
Province/territory	33 (48.5) from Ontario
Age	
Under 12 months	14 (20.6)
1–2 years	14 (20.6)
3–5 years	11 (16.2)
6–12 years	13 (19.1)
12–17 years	16 (23.5)
Sex	33 (48.5) female
Diagnosed with:	
Food allergies	56 (82.4)
Eczema	36 (52.9)
Asthma	16 (23.5)
Hayfever	7 (10.3)
Mode of exposure triggering reaction	
Ingestion	68 (100)
Inhalation	6 (8.80)
Contact	11 (16.2)
Length of time after exposure to symptoms	
Less than 2 h	56 (82.4)
2 h or more	10 (14.7)
Unknown	2 (2.90)
Length of time from exposure to resolution	
Less than 48 h	49 (72.1)
48 h or more	18 (26.5)
Unknown	1 (1.40)
Any medication given	
Epinephrine	13 (19.1)
Antihistamines	45 (66.2)
Oral steroids	12 (17.7)
Other	4 (5.90)
None	18 (26.5)
Sought medical attention	
Emergency Department—own transport	19 (27.9)
Emergency Department—by ambulance	8 (11.8)
Family practitioner/walk-in clinic	13 (19.1)
Other	1 (1.50)
No medical attention	30 (44.1)
Food caused by packaged product	38 (55.9)

The most common emerging allergens reported were fruits/vegetables (58.8%), seeds (22.1%), legumes (19.1%), and other (11.8%) (Table 2). Parents of children < 6 years were 5.3 times more likely to report an allergic reaction to legumes, compared to parents of children ≥ 6 years (p = 0.01). 38.5% of reported legume allergic children also had a diagnosed peanut allergy. More than half (55.9%) of patients reported that a packaged product caused the reaction, but only one patient reported possible cross-contamination with a known allergen (1.47%).

**Table 2:** Most common emerging allergens and allergist ranking of possible or probable IgE-mediated allergy

Food allergen type	Number of patients reporting one or more foods from a specific food group caused the allergic reaction (%)	Foods^a^ from each group reported as being responsible for the allergic reaction, n	Allergist ranked as “Possible or probable” IgE-mediated allergy, n (% of total reported)
Legumes	13 (19.1)	Green pea: n = 13	Green pea: n = 9 (69.2)
		Lentil: n = 7	Lentil: n = 4 (57.1)
		Chickpeas: n = 3	Chickpeas: n = 3 (100)
		Split peas: n = 1	Split peas: n = 1 (100)
		Lupin: n = 1	Lupin: n = 1 (100)
Seeds	15 (22.1)	Coconut: n = 7	Coconut: n = 3 (42.9)
		Pumpkin seed: n = 4	Pumpkin seed: n = 1 (25.0)
		Chia seeds: n = 3	Chia seeds: n = 1 (33.3)
		Poppy seed: n = 2	Poppy seed: n = 0 (0.00)
		Flax: n = 2	Flax: n = 1 (50.0)
		Sunflower: n = 1	Sunflower: n = 1 (100)
		Hemp seeds: n = 1	Hemp seeds: n = 1 (100)
Fruits & vegetables	40 (58.8)	Corn: n = 9	Corn: n = 1 (11.1)
		Pineapple: n = 6	Pineapple: n = 5 (83.3)
		Mango: n = 5	Mango: n = 1 (20.0)
		Banana: n = 4	Banana: n = 1 (25.0)
		Kiwi: n = 3	Kiwi: n = 2 (67.7)
		Apple: n = 3	Apple: n = 0 (0.00)
		Orange: n = 3	Orange: n = 1 (33.3)
		Garlic: n = 2	Garlic: n = 1 (50.0)
		Strawberry: n = 2	Strawberry: n = 1 (50.0)
		Tangerine: n = 2	Tangerine: n = 1 (50.0)
		Sweet potato: n = 2	Sweet potato: n = 0 (0.00)
		Avocado: n = 2	Avocado: n = 0 (0.00)
		Pear: n = 1	Pear: n = 0 (0.00)
		Carrots: n = 1	Carrots: n = 0 (0.00)
		Dragonfruit: n = 1	Dragonfruit: n = 0 (0.00)
		Broccoli: n = 1	Broccoli: n = 0 (0.00)
		Cauliflower: n = 1	Cauliflower: n = 0 (0.00)
		Raspberry: n = 1	Raspberry: n = 0 (0.00)
		Blueberry: n = 1	Blueberry: n = 0 (0.00)
		Bell peppers: n = 1	Bell peppers: n = 1 (100)
		Peach: n = 1	Peach: n = 0 (0.00)
		Squash: n = 1	Squash: n = 0 (0.00)
		Ginger: n = 1	Ginger: n = 0 (0.00)
		Onion: n = 1	Onion: n = 0 (0.00)
		Grapes: n = 1	Grapes: n = 0 (0.00)
		Tomato: n = 1	Tomato: n = 0 (0.00)
		Zucchini: n = 1	Zucchini: n = 1 (100)
		Potato: n = 1	Potato: n = 0 (0.00)
Other	8 (11.8)	Beef: n = 2	Beef: n = 0 (0.00)
		Rice: n = 2	Rice: n = 0 (0.00)
		Oats: n = 2	Oats: n = 1 (50.0)
		Buckwheat: n = 2	Buckwheat: n = 2 (100)
		Artificial red food	Artificial red food
		colouring: n = 1	colouring: n = 0 (0.00)
		Lamb: n = 1	Lamb: n = 0 (0.00)
		Poultry: n = 1	Poultry: n = 0 (0.00)
		Coffee: n = 1	Coffee: n = 0 (0.00)
		Lecithin: n = 1	Lecithin: n = 0 (0.00)
		Pepper: n = 1	Pepper: n = 0 (0.00)

^a^Patients often reported more than one food as being responsible for the allergic reaction described in their survey, so the numbers in this column may add up to more than the number of patients reporting the allergic reaction. For example, some patients reported green pea alone as being responsible for their reaction, whereas some reported both green pea and lentil

**Fig. 1:**
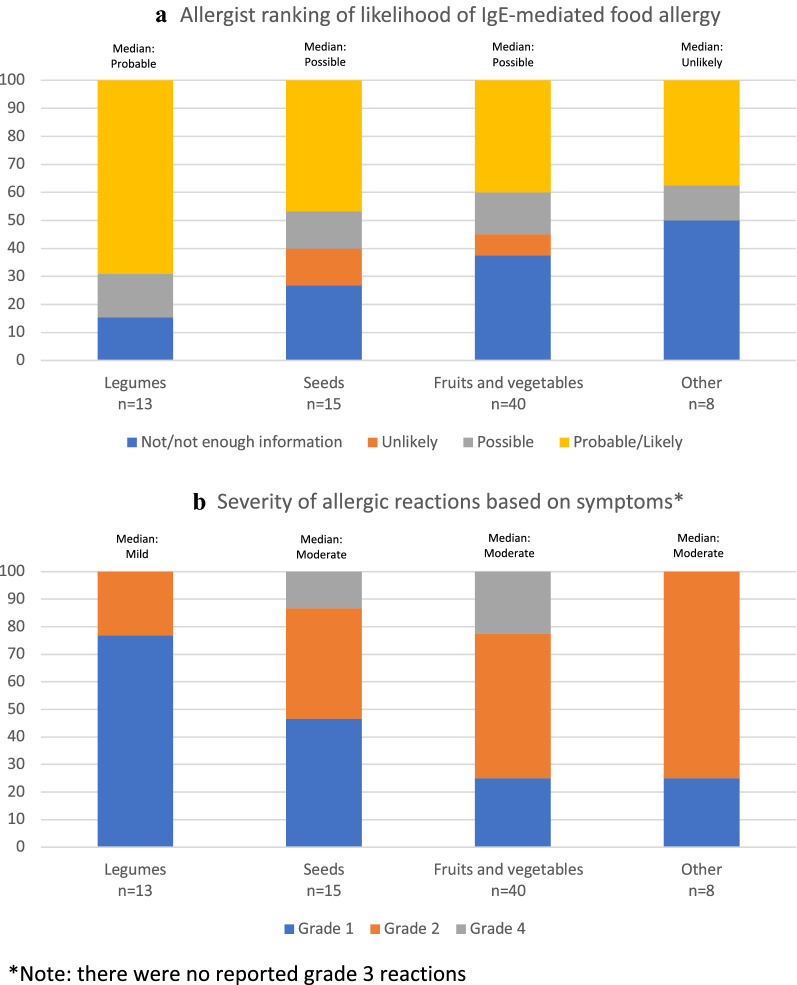
**a** Allergist ranking of likelihood of IgE-mediated food allergy. **b** Severity of allergic reactions based on symptoms*. *Note: there were no reported grade 3 reactions

Of the 68 participants, 20 (29.4%) were classified as ‘Not/Not enough information,’ 5 (7.4%) were ‘Unlikely,’ 11 (16.2%) were ‘Possible,’ and 32 (47.0%) were ‘Probable/Likely’ IgE-mediated food allergy. The allergist ranking by food allergen type is depicted in Fig. 1a.

Twenty-five (36.8%) children experienced a grade 1 reaction, 33 (48.5%) a grade 2 reaction, and 10 (14.7%) a grade 4 reaction (Fig. 1b). Thirteen (19.1%) patients received epinephrine for their allergic reaction. There was a statistical association between a greater reaction severity and likelihood of receiving epinephrine (p = 0.0021), but there was no association between epinephrine use and food allergen type.

This is the first study to collect information from Canadian parents regarding their child’s allergic reactions to foods beyond the 11 Health Canada priority allergens. We found that fruits/vegetables are the most commonly reported emerging allergen, followed by seeds, and legumes. Additionally, we found that reported reactions were milder for legumes than for fruits/vegetables, seeds, and others (median mild vs. moderate symptoms). However, allergists ranked legumes and seeds as probable/likely allergens more than the other categories.

Prior research has often dismissed reactions to fruits/vegetables as being associated with pollen-food syndrome, characterized by mild, transient oropharyngeal symptoms due to sensitization to the profilin protein [3]. Our allergist ratings support this finding, with nearly 50% of the patients reporting fruit/vegetable allergies being classified as ‘not’ or ‘unlikely to be’ a systemic IgE-mediated allergic reaction. In some European countries, fruits/vegetables have been associated with more significant anaphylactic symptoms due to sensitization to lipid transfer proteins [4]. A recent review points to a growing number of reports of anaphylaxis to fruits/vegetables [3] , which is in line with our data noting 74.4% of patients with grade 2 symptoms or higher. A hypothesis for this increase in anaphylaxis is the increasing popularity of a variety of fruits/vegetables in the diet, especially in their concentrated form in juices and smoothies [3].

A similar (although less pronounced) trend was observed for seeds, with allergists ranking ‘possible’ even though more than half of the patients had a grade 2 or 4 reaction. Coconut, which was the most common of the seeds, appears to be following a similar trend as fruits/vegetables, with increasing consumption in the form of coconut milk, and increases in use of coconut-based skin care products, which is an important concern in infants with atopic dermatitis who are at high risk of sensitization via the skin [5].

It was interesting that allergists had more confidence that reactions to legumes were consistent with IgE-mediated food allergy, even though close to two-thirds of these reactions were associated with grade 1 symptoms only. Legumes other than peanut are an important food group causing IgE-mediated allergic reactions in Canadian children, especially in children under the age of 6. This is in line with Spanish data, noting legumes are the 5th most common food allergen among children < 5 years, likely due to legumes being an important part of the diet in that country [6]. With the pervasiveness of veganism in Canada and the increasing use of legume proteins (i.e. pea protein) in foods [7, 8], it is not surprising that allergic reactions to legumes were commonly reported in our study.

Our study design was limited in that we were unable to determine which food(s) caused the reaction if the patient also reported a diagnosed food allergy to a priority allergen. However, more than half of the patients reported a reaction to a packaged food which listed the emerging allergen as an ingredient. Inclusion of the ingredient list increased our confidence that the patient reacted to the food listed and not a priority allergen. We attempted to confirm whether there was cross-contamination with a priority allergen, but only one patient obtained that information (which was negative). In addition, our study was initially designed to collect diagnostic testing from allergists to support parent reports, but no test results were received. Therefore, two allergists reviewed the parent surveys and rated the probability of food allergy. Slight differences arose based on the allergists’ interpretation of the likelihood categories, differential prioritization of clinical factors (i.e., symptoms, treatment), and clinical experience, in evaluating whether a reaction is IgE-mediated or not. To address this, we assigned the likelihood category with the lowest certainty between the two allergists to obtain a more conservative estimate, and additionally included the results from our objective reaction grading system. Further, allergists were not blinded to the culprit food, which could have biased the results. However, not blinding the allergists is also a strength, because there is a much stronger literature base for certain allergens (i.e. peas) being a systemic IgE-mediated food allergy than other allergens (i.e. corn). It is important that the allergist take existing literature into account when ranking.

Our study highlights that non-priority food allergens, namely legumes and seeds, can lead to probable/likely allergic reactions in Canadian children. We predict pea protein in particular will cause problems for patients (with or without peanut allergy), as it is increasing in the food supply and there have been other recent reports of pea anaphylaxis in Canada [9].

However, more research is needed to confirm reports of reactions to emerging allergens, and to document their inclusion as ingredients in packaged foods. Protocols for immunotherapy to treat allergies to these emerging allergens will be needed if they become more common. Lastly, the importance of early introduction to prevent development of food allergy should be considered as these foods become more common in an infant’s environment.

## Data Availability

The datasets generated and/or analysed during the current study are not publicly available.
